# Emphysema is associated with thoracic vertebral bone attenuation on chest CT scan in HIV-infected individuals

**DOI:** 10.1371/journal.pone.0176719

**Published:** 2017-04-27

**Authors:** Alycia Petraglia, Joseph K. Leader, Matthew Gingo, Meghan Fitzpatrick, John Ries, Cathy Kessinger, Lorrie Lucht, Danielle Camp, Alison Morris, Jessica Bon

**Affiliations:** 1 Department of Medicine, University of Pittsburgh, Pittsburgh, Pennsylvania, United States of America; 2 Department of Radiology, University of Pittsburgh, Pittsburgh, Pennsylvania, United States of America; 3 Department of Medicine, Division of Pulmonary, Allergy & Critical Care Medicine, University of Pittsburgh, Pittsburgh, Pennsylvania, United States of America; 4 Department of Immunology, University of Pittsburgh, Pittsburgh, Pennsylvania, United States of America; University of Alabama at Birmingham, UNITED STATES

## Abstract

**Background:**

Age-related chronic diseases are prevalent in HIV-infected persons in the antiretroviral therapy (ART) era. Bone mineral density (BMD) loss and emphysema have separately been shown to occur at a younger age and with lesser risk exposure in HIV-infected compared to HIV-uninfected individuals. In non-HIV infected smokers, emphysema has been shown to independently predict low BMD. We hypothesized that emphysema would independently associate with thoracic vertebral bone attenuation, a surrogate for bone mineral density, in HIV-infected individuals.

**Methods:**

Clinical, pulmonary function, and radiographic data were analyzed for 164 individuals from the University of Pittsburgh’s HIV Lung Research Center cohort. Chest CT scans were used to quantify emphysema and compute Hounsfield Unit (HU) attenuation of the 4^th^, 7^th^, and 10^th^ thoracic vertebrae. The association between mean HU attenuation values across the three vertebrae and radiographic emphysema, age, sex, body mass index (BMI), steroid use, viral load, CD4 count, and forced expiratory volume in the first second (FEV1) was assessed by univariate and multivariate analyses.

**Results:**

In univariate analysis, mean HU attenuation decreased with increasing age (p<0.001), pack years (p = 0.047), and percent emphysema (p<0.001). In a multivariable model, including pack years, age, sex, ART and steroid use, greater emphysema was independently associated with this surrogate marker of BMD in HIV-infected individuals (p = 0.034).

**Conclusions:**

The association of emphysema with thoracic bone attenuation in HIV-infected individuals is consistent with previous reports in non-HIV infected smokers. These findings suggest that emphysema should be considered a potential marker of osteoporosis risk in HIV-infected individuals.

## Introduction

The widespread introduction of highly effective antiretroviral therapy (ART) for human immunodeficiency virus (HIV) infection has dramatically decreased HIV-associated mortality [1]. Consequently, the prevalence of chronic diseases, such as COPD and osteoporosis, has increased and occurred prematurely in the HIV-infected population [1–6]. Low bone mineral density (BMD) is a metabolic condition with increasing relevance in HIV-infected patients, with an estimated incidence that is three-fold greater than in the general HIV-uninfected population [3–4,7–9]. HIV-infected individuals carry a five-fold increased risk of hip fracture [4] and experience significantly greater annual BMD loss [9–10]. In addition to risk factors shared with the general population, such as age, sex, and low body mass index (BMI), studies have shown that factors unique to HIV, including ART, viral load, and inflammatory mediators, contribute to bone demineralization [4,7–8].

Chronic obstructive pulmonary disease (COPD) is associated with low BMD and greater fracture risk in non-HIV infected smokers [11–12]. The presence of emphysema alone on CT imaging is a strong, independent predictor of low BMD in this group [13–14]. Obstructive lung disease is increasingly recognized as a common comorbidity in individuals infected by HIV [15] and HIV-related factors appear to independently contribute to the development of COPD [15–18]. Emphysema has been shown to occur at an earlier age with less tobacco exposure in HIV-infected smokers [5–6,16–17] and may likewise be related to low BMD in persons with HIV infection. While the impact of multimorbidity on quality of life and survival in HIV infection has been investigated [19–21], research efforts exploring the link between lung and bone disease are lacking.

HIV management guidelines recommend osteoporosis screening with baseline dual x-ray absorptiometry (DXA) BMD assessment in all HIV-infected postmenopausal women and in men at age 50 [22]. The presence of emphysema may accelerate BMD loss and may place HIV-infected individuals who smoke that are at an even greater risk of fracture. To evaluate novel factors associated with low BMD in individuals with HIV-related lung disease, we assessed the relationship between emphysema and thoracic vertebral bone attenuation, a surrogate marker of BMD, depicted on chest CT images in a cohort of HIV-infected smokers.

## Methods

### Subject selection

The study population consisted of 164 subjects selected from the HIV Lung Research Center cohort (HLRC) at the University of Pittsburgh who had chest CT scans. The HLRC cohort is comprised of HIV-infected males and females 18 years of age or older who were recruited from the University of Pittsburgh Medical Center HIV/AIDS clinic. Recruitment for the HLRC cohort occurred between July 1, 2007 and May 15, 2009. Subject selection was made without any a priori knowledge of BMD or lung function. Available clinical data for this cohort included demographic data (age, sex, race, height, weight), medication history (oral steroid use, antiretroviral therapy), smoking history data, chest CT scans, pulmonary function data, and HIV viral load and CD4 counts. Participant sex refers to self-reported biological sex. The study protocol was approved by the University of Pittsburgh Institutional Review Board and written informed consent was obtained for each subject.

### CT examination

Non-contrast chest CT examinations were performed on a General Electric (GE) LightSpeed VCT (64-detector) (Buckinghamshire, UK) scanner at a radiation exposure of 100–120 mAs. Contiguous CT images were reconstructed using both a low spatial frequency kernel (e.g. GE’s “standard” kernel) at 0.625 mm and 2.5 mm thicknesses. A single blinded image analyst performed the segmentation and analysis of the lung and thoracic vertebrae depicted on CT images. The lung parenchyma was automatically segmented from the chest wall and mediastinum using in-house software. A density mask approach was used to quantify the percentage of low attenuation areas associated with emphysema (LAA%) using a threshold of -950 Hounsfield units (HU). Using a previously validated approach [23–24], a semi-circular region of interest (ROI) was manually drawn in the central region of the T4, T7, and T10 thoracic vertebrae excluding cortical bone and the posterior basivertebral venous plexus. Mean HU value across the three vertebral ROIs was computed as a surrogate for BMD [23, 24].

### Statistical analysis

Differences in thoracic vertebral bone attenuation by sex, ART use, and systemic steroid use were assessed by student’s t-test and Wilcoxon signed rank test for normally and non-normally distributed variables respectively. The difference in mean thoracic vertebral bone attenuation between quartiles of LAA% was assessed by Kruskall Wallis test. The association between mean thoracic vertebral bone attenuation and LAA%, age, BMI, HIV viral load, CD4 count, and lung function (FEV1) was assessed by univariable generalized linear modeling. LAA% was log-transformed because of its highly-skewed distribution. The relationship between bone attenuation and LAA%, adjusted for established osteoporosis risk factors and covariates significant in univariable analysis, was assessed using generalized linear modeling. All statistical analyses were performed with SAS 9.2 and Stata 13.1.

## Results

### Subject characteristics

The mean age of the cohort was 45.1 years, and consisted of a greater number of males than females (Table 1). Although the majority of participants were former or current smokers, less than 20% had evidence of airflow obstruction. Most participants were taking ART, with tenofovir disoproxil fumarate (TDF) use in 82.6%, and, as expected, median viral load copy numbers were low and median CD4 counts high. Only a small proportion of participants reported systemic steroid use within the past twelve months. Over half of the cohort was overweight or obese, and none were underweight. Fifty-three participants had Vitamin D levels measured within one year of their CT scan. The mean Vitamin D level was 26.0 ng/mL (SD 12.9 ng/mL), and of these participants, 18 (34%) had a level which was insufficient (below 20 ng/mL).

**Table 1:** Subject characteristics (n = 164).

Age, mean years, SD [range]	45.1 ± 10.2 [20–71]
**Age 20–40 years, n (%)**	43 (26.2%)
**Age 41–60 years, n (%)**	112 (68.3%
**Age > 60 years, n (%)**	9 (5.5%)
**Male, n (%)**	115 (70.1%)
**BMI, mean, SD [range]**	27.7 ± 6.5 [18.8–53.8]
**BMI <18.5 underweight, n (%)**	0 (0%)
**BMI 18.5–25 normal, n (%)**	65 (39.6%)
**BMI 25–30 overweight, n (%)**	55 (33.6%)
**BMI > 30 obese, n (%)**	44 (26.8%)
**Race**	
**Caucasian, n (%)**	71 (43.3%)
**Black, n (%)**	93 (56.7%)
**Smoking status**	
**Never, n (%)**	30 (18.4%)
**Current, n (%)**	96 (58.9%)
**Former, n (%)**	37 (22.7%)
**Pack years, mean, SD [range]**	17.3 ± 17.6 [0–105]
**Viral load, median copies/mL [IQR]**	49 [630]
**CD4 count, median cells/microL [IQR]**	561.5 [369]
**Overall Antiretroviral therapy (yes), n (%)**	132 (90%)
**TDF use, n (%)**	109 (82.6%)
**Oral steroid use last 12 months (yes), n (%)**	9 (5.7%)
**Estrogen/progesterone use, n (%)**	3 (1.8%)
**Testosterone use, n (%)**	1 (0.6%)
**FEV1 mean percent predicted, SD**	94.2 ± 17.9
**Obstruction**	
**At Risk, n (%)**	136 (82.93%)
**GOLD I, n (%)**	14 (8.54%)
**GOLD II, n (%)**	12 (7.31%)
**GOLD III, n (%)**	2 (1.22%)
**Attenuation area %, median [IQR]**	6.9 [11.7]
**Mean HU value across T4, T7, T10, SD**	212.8 ± 46.4

SD = standard deviation, IQR = interquartile range, FEV1 = forced expiratory volume in the first second, Emphysema = <-950 HU on chest ct

### Factors associated with thoracic vertebral bone attenuation

Thoracic vertebral bone attenuation was not significantly different between male and female participants (208.4 ± 6.3 HU [95% CI 195.9–221.0] males vs. 214.6 ± 4.4 HU [95% CI 205.8–223.4] females, p = 0.44). Steroid use did not impact bone attenuation, but participants on ART had lower thoracic vertebral bone attenuation compared to those cohort participants not on ART (207.7 ± 8.8 HU [95% CI 199.6–214.9] vs. 235.4 ± 3.9 [95% CI 217.9–252.7], p = 0.003). Participants with higher quartiles of LAA% on chest CT scan had lower thoracic vertebral bone attenuation (quartile 1: 233.2 ± 49.8, quartile 2: 216.0 ± 42.8, quartile 3: 200.8 ± 45.2, quartile 4: 200.2 ± 41.1; p = 0.007).

In univariable analysis, thoracic vertebral bone attenuation decreased with increasing age (r^2^ = 0.18, <0.001), greater pack years (r^2^ = 0.03, p = 0.047), and increasing LAA% (r^2^ = .10, p<0.001). Thoracic vertebral bone attenuation was not associated with FEV_1_, DLCO, viral load, CD4 count, or body mass index. In a multivariable model, including pack years, age, sex, ART and oral steroid use in the past 12 months, greater emphysema (p = 0.034) was independently associated with lower thoracic vertebral density in HIV-infected individuals (Table 2). When ART was replaced with TDF use in multivariable modeling, the relationship between LAA% and thoracic vertebral bone attenuation remained significant (p = 0.04).

**Table 2:** Factors associated with thoracic vertebral bone attenuation, multivariable analysis.

	Beta Coefficient (95% confidence interval)	p-Value
Attenuation Area %*	-8.0 (-15.4, -0.9)	0.034
**Age per year**	-1.3 (-2.1, -0.50)	0.002
**Sex (male)**	-4.8 (-20.4 10.7)	0.54
**Pack Years**	-0.15 (-0.56, 0.25)	0.46
**Anti-retroviral therapy (yes)**	-2.5 (-22.7, 17.7)	0.81
Oral steroids**	13.4 (-15.5, 42.3)	0.36

*Log transformed values used

**Oral steroids = any use in the past 12 months

## Discussion

In a well-characterized cohort of both HIV-infected men and women, we found that greater quantitative emphysema on CT scan was associated with lower thoracic vertebral bone attenuation. These findings were independent of age, airflow obstruction, and other traditional osteoporosis risk factors. A previous study comparing thoracic vertebral bone attenuation on chest CT imaging with DXA measurements of hip and lumbar spine BMD in individuals with COPD found a strong correlation between thoracic vertebral bone attenuation and DXA BMD measurements [23], suggesting that CT bone attenuation is a surrogate for bone health. A receiver-operating characteristic curve analysis in this same study revealed a high sensitivity (93%) and specificity (97%) for osteoporosis using a threshold of 147 HU, which would classify 5.5% of our HIV-infected cohort, 8.2% of female participants and 4.3% of male participants, as having osteoporosis. Whereas emphysema has been associated with low BMD across multiple cohorts of non-HIV infected smokers [13–14,23–24], our study is the first to establish a link between radiographic emphysema and a surrogate for BMD in HIV infected individuals.

Our finding of an association between radiographic emphysema and thoracic vertebral bone attenuation, a surrogate for BMD, in HIV suggests that the presence of emphysema on CT imaging may serve as a marker of low BMD in HIV-infected individuals. An increased prevalence of early emphysema and airflow obstruction and accelerated bone mineral density loss has been well-documented in HIV-infected individuals. Gingo and colleagues have previously shown a high prevalence of respiratory symptoms, diffusion impairment, and persistent airway obstruction in individuals with HIV infection during the post ART era [18]. In an analysis between HIV-infected and HIV-uninfected individuals, the rate of incident COPD was 8% higher in HIV-infected persons 50 years of age or older and 17% higher in HIV-infected persons younger than age 50 compared to HIV-uninfected individuals after controlling for age, race and ethnicity, sex, alcohol use, drug abuse, and hepatitis C infection [6], suggesting an earlier onset of lung disease with less tobacco exposure in HIV-infected persons. Osteoporotic fractures are likewise more prevalent in HIV-infected individuals compared to the general population [3–4,7–9]. Pre-menopausal HIV-infected woman have a significantly higher fracture rate compared to women without HIV infection and, in general, patients with HIV infection have a 75% higher risk of all clinical fractures as well as a five-fold increase in hip fracture occurrence [4,25]. Both HIV infection and obstructive lung disease have been associated with higher mortality following hip fracture, suggesting that earlier baseline assessment of BMD in HIV-infected smokers should be considered [1–4,7–9,26–27].

The association of emphysema and low BMD is suggestive of a common pathogenesis linking parenchymal destruction and bone loss in HIV infection. Although multiple factors likely contribute to accelerated BMD loss and increased emphysema in HIV-infected individuals, exact mechanisms remain unclear [3, 7–9]. Prolonged ART has been hypothesized to contribute to the higher incidence and more rapid progression of BMD loss in HIV-infected patients [3–4], and we did find that participants on ART had lower thoracic bone attenuation compared to those participants not receiving therapy. Yet, data on the role of specific mechanisms of ART drugs in bone demineralization remain controversial [3–4, 7–8]. Accelerated aging has been implicated in the pathogenesis of emphysema and other noninfectious comorbidities, including osteoporosis in HIV infection, and may offer a mechanistic link between these disease processes [1, 28, 29]. Immuno-inflammatory phenomena implicated in the normal aging process, including immune senescence, depreciation of the adaptive immune system, and heightened systemic inflammation, are also pathophysiologic sequalae of HIV infection, suggesting HIV infection can potentiate the biological mechanisms of aging [28]. As in aging, HIV infection is characterized by enhanced inflammation, generally in the context of persistent immune activation [29–32]. Fitzpatrick and colleagues found relationships between peripheral T-lymphocyte activation and senescence, the classic inflammatory markers (IL)-6 and C-reactive protein, and PFT abnormalities in HIV [29, 33]. More recently, they showed that baseline measures of monocyte activation and endothelial dysfunction are associated with lower pulmonary function in HIV-infected persons [29]. Further investigation of common effector cells, converging inflammatory pathways, and immunologic parallels responsible for lung parenchymal destruction and BMD loss should shed light on the link between emphysema and skeletal abnormalities in HIV infection.

Our study had several strengths. The availability of a well-characterized cohort of individuals with HIV infection provided the opportunity to examine the relationship between HIV-related lung disease and bone health while accounting for many factors traditionally associated with osteoporosis. The severity of lung disease within the cohort was mild, thus eliminating confounders, including steroid use or cachexia, which may be present in HIV-infected individuals with more severe underlying lung disease. Whether the results of this study are generalizable to HIV-infected individuals with greater lung impairment is unclear and will need to be validated in a separate, more affected cohort.

This study included several limitations. First, Vitamin D levels were not measured systemically in each participant. Vitamin D deficiency appears to be an under-recognized and underdiagnosed disorder in the general population. Adequate Vitamin D levels have already been established as paramount for bone health, and deficiency has also been shown to be exceedingly prevalent in chronic lung disease [34]. Perhaps Vitamin D deficiency could have contributed to both bone loss and lung disease. DXA imaging, the generally accepted standard assessment of BMD, was not available for most cohort participants. However, the use of CT image analysis of thoracic vertebrae as a marker of bone health has been investigated in a prior non-HIV infected COPD cohort and reportedly provides a reliable, and readily accessible, surrogate of BMD [14, 17, 23]. We did not apply a pre-defined threshold to determine the presence of emphysema on CT scan as there is no universally validated “emphysema” threshold and our CT analysis was limited to quantitative, rather than semi-quantitative visual, assessment alone. Prior studies in non-HIV infected cohorts have demonstrated an association of both semi-quantitative visual emphysema [13] and quantitative emphysema [14] with bone mineral density. We chose to limit this study to emphysema assessed by quantitative methods since this technology is automated, widely available, and does not require specialized training to grade emphysema severity based on a semi-quantitative visual scoring system.

## Conclusion

In conclusion, we are the first to recognize that radiographic emphysema independently associates with a surrogate of BMD in HIV-infected individuals. Our findings suggest that emphysema on CT imaging, independent of other osteoporosis risk factors, should be considered in studies examining mechanisms of bone loss in HIV infection. The findings from this study support the need for future studies to understand the mechanisms linking bone loss and lung parenchymal destruction in both HIV-infected and uninfected smokers.

## Supporting information

S1 Table Cohort Data. (XLSX)
